# Spastin-Interacting Protein NA14/SSNA1 Functions in Cytokinesis and Axon Development

**DOI:** 10.1371/journal.pone.0112428

**Published:** 2014-11-12

**Authors:** Uma Goyal, Benoît Renvoisé, Jaerak Chang, Craig Blackstone

**Affiliations:** Cell Biology Section, Neurogenetics Branch, National Institute of Neurological Disorders and Stroke, National Institutes of Health, Bethesda, Maryland, United States of America; Cambridge University, United Kingdom

## Abstract

Hereditary spastic paraplegias (HSPs) are a genetically diverse group of inherited neurological disorders (SPG1-72) with the cardinal feature of prominent lower-extremity spasticity due to a length-dependent axonopathy of corticospinal motor neurons. The most frequent form of autosomal dominant HSP results from mutations of the *SPG4* gene product spastin. This is an ATPase associated with diverse cellular activities (AAA) protein that binds to and severs microtubules. While spastin participates in crucial cellular processes such as cytokinesis, endosomal tubulation, and axon development, its role in HSP pathogenesis remains unclear. Spastin interacts in cells with the NA14 protein, a major target for auto-antibodies in Sjögren's syndrome (nuclear autoantigen 1; SSNA1). Our analysis of endogenous spastin and NA14 proteins in HeLa cells and rat cortical neurons in primary culture revealed a clear distribution of both proteins to centrosomes, with NA14 localizing specifically to centrioles. Stable NA14 knockdown in cell lines dramatically affected cell division, in particular cytokinesis. Furthermore, overexpression of NA14 in neurons significantly increased axon outgrowth and branching, while also enhancing neuronal differentiation. We postulate that NA14 may act as an adaptor protein regulating spastin localization to centrosomes, temporally and spatially regulating the microtubule-severing activity of spastin that is particularly critical during the cell cycle and neuronal development.

## Introduction

Hereditary spastic paraplegias (HSPs) are a large, genetically diverse group of neurological disorders characterized by prominent lower extremity spasticity, resulting from a distal axonopathy of corticospinal motor neurons [1]–[4]. The HSPs have historically been divided into two broad categories, ‘pure’ or ‘complicated’, based on the presence (complicated) or absence (pure) of associated clinical features such as cognitive dysfunction, distal amyotrophy, thin corpus callosum, white matter abnormalities, and neuropathy [2]–[5]. More recently, a genetic classification scheme has taken hold, with over 70 distinct loci mapped (SPG1-72). Greater than 50 HSP genes and their protein products have now been identified, and sequence analyses and published studies support pathogenic convergence within a number of common cellular themes, including alterations in endoplasmic reticulum (ER) network shaping/distribution, lipid/cholesterol metabolism, mitochondrial function, myelination, bone morphogenetic protein signaling, protein/membrane trafficking, autophagy, and endo-lysosomal function [1], [2], [6], [7].

SPG4 is by far the most common form of autosomal dominant HSP, accounting for about 40% of cases. It is caused by mutations in the *SPAST* gene that encodes the spastin protein [8], a member of the AAA (ATPase associated with diverse cellular activities) protein family that binds to and severs microtubules [9]. Spastin exists in 4 isoforms generated through the use of different translation initiation sites, commencing at methionine residues 1 (M1 isoform; 68 kDa) or 87 (M87 isoform; 60 kDa), along with alternative mRNA splicing of exon 4 that generates two additional, smaller forms [10]. Spastin M1 is an integral membrane protein harboring an N-terminal intramembrane hairpin that localizes to ER and endosomes, while the smaller spastin M87 isoform is present in the cytoplasm as well as at endosomes, centrosomes, midbodies, and spindle poles [11]–[14].

Spastin interacts with the centrosomal protein NA14, a major btarget for auto-antibodies in Sjögren's syndrome (nuclear autoantigen 1, SSNA1) [14]–[16]. The functions and clinical relevance of this NA14 interaction for HSP pathogenesis remain largely unknown. NA14 is a small protein (∼13 kDa) that is able to adopt a highly helical, coiled-coil structure, and it is predicted to be a distant homolog of the actin-binding protein tropomyosin. NA14 can assemble into large fibrils, and this higher-order structure is important for its interaction with spastin as well as for microtubule dynamics; it may also contribute to its immunogenicity [17].

Orthologs of NA14 appear to be lacking in widely-studied eukaryotic model organisms such as *S. cerevisiae*, *C. elegans*, and *D. melanogaster*, but they are present in the flagellated green alga *C. reinhardtii*, trematode worms, and protozoan parasites [17], [18]. NA14 localizes to primary cilia, basal bodies, centrosome, and plasma membrane. Furthermore, studies have shown that the *C. reinhardtii* ortholog of NA14, Deflagellation Inducible Protein 13 (DIP13), has a similar cellular distribution to mammalian NA14; DIP13 localizes to microtubules in the flagella axoneme, basal bodies and cytoplasm [18], [19]. Knock down of DIP13 expression results in cells having multiple nuclei and flagella [18], and overexpression of DIP13 causes a defect in assembly of full-length flagella [19].

In mammalian cells, microtubules are nucleated from the centrosome, an organelle that serves as the principal microtubule-organizing center in cells and which is crucial for maintaining cell polarity and forming the mitotic spindle during cell division [20]. It comprises a pair of centrioles embedded within an amorphous protein mass termed the pericentriolar material [21], [22]. The pericentriolar proteins, including γ-tubulin and pericentrin, are responsible for microtubule nucleation and anchoring [23].

Centrosomes influence a large number of microtubule-related processes including intracellular trafficking, cell morphology, and motility. During mitosis, centrosomes direct the formation of bipolar spindles to segregate chromosomes and also release central microtubules from the midbodies to completion cell division. In neurons, the centrosome determines polarity [24] and plays a role in neurogenesis, neuronal migration, and neuronal differentiation [25]. The centrosome is generally positioned at the base of the nascent axon to initiate growth, and the coordination between actin- and centrosome-dependent microtubule growth is likely a key determinant of axon extension. Growing axons also require a steady delivery of membrane and microtubules to the migrating growth cone. Thus, proper positioning of the centrosome is crucial for membrane trafficking and polarized microtubule-based delivery to the axon [26], [27].

Here, we have tested our hypothesis that NA14 plays a role in axonal development. We found that, like spastin, NA14 enhances the formation of axons. Endogenous spastin and NA14 proteins in HeLa cells and rat cortical neurons in primary culture show a clear distribution to centrosomes, with NA14 specifically at centrioles. Stable knockdown of NA14 dramatically affects cell division, in particular cytokinesis. Furthermore, overexpression of NA14 in neurons significantly increases axon outgrowth and branching; it also enhances neuronal differentiation without modifying the number of centrosomes. Taken together, our data suggest that NA14 may act as adaptor protein, regulating spastin localization to centrosomes and possibly contributing to the spatial and temporal regulation of its microtubule-severing activity.

## Materials and Methods

### DNA constructs

Complementary DNAs comprising the coding sequences of human M1 and M87 spastin (GenBank NM_014946, including the exon 4 splice cassette) were cloned into the mammalian expression vector pGW1-Myc, which contains an N-terminal Myc-epitope tag [11], [28]. Human full-length NA14 (GenBank NP_003722.1) and a Δ1–32 amino acid deletion form were cloned into the *Eco*RI site of pGW1-HA, which harbors an N-terminal hemagglutinin (HA)-epitope tag. For producing lentivirus, wild-type NA14 cDNA was cloned into the pCDH-CMV-MCS-EF1-Puro lentiviral vector (Systems Biosciences, Mountain View, CA, USA); sequences encoding HA and EGFP epitopes were cloned into *Xba*I and *Eco*RI sites. To establish NA14-depleted cell lines, shRNA sequences specific for NA14 or else a control sequence (CTL) were cloned into the pLKO.1 Puro vector (Addgene, Cambridge, MA, USA): shCTL, 5′-GCAATCGAAGCTCGGCTAC-3′; shNA14-1, 5′-ACAACAACGAGCTGGTCAA-3′; shNA14-2, 5′-AGGAGGAGGAGGACGAGAA-3′; and shNA14-3, 5′-AGGAGGAGGACGAGAAGCA-3′. All constructs were confirmed by DNA sequencing.

### Antibodies

An affinity-purified anti-spastin polyclonal rabbit antibody, used at 1∶1000 dilution, was described previously [29]. The following antibodies were obtained commercially: mouse monoclonal anti-spastin (1∶1000; IgG_2a_, clone Sp 6C6, Sigma-Aldrich, St. Louis, MO, USA), mouse polyclonal anti-SSNA1 (1∶500; Abnova, Taipei, Taiwan), rabbit polyclonal anti-SSNA1 (1∶1000; Proteintech Group, Chicago, IL, USA), mouse monoclonal anti-c-Myc epitope (1∶1000; IgG_1_, clone 9E10, Santa Cruz Biotechnology, Dallas, TX, USA), goat polyclonal anti-c-Myc epitope (1∶500; A190-104A, Bethyl Laboratories, Montgomery, TX, USA), mouse monoclonal anti-HA probe (1∶1000; IgG_2a_, clone F-7, Santa Cruz Biotechnology), rabbit polyclonal anti-HA probe (1∶1000; ab9110-100, Abcam, Cambridge, MA, USA), mouse monoclonal anti-α-actin (1∶1000; clone AC-40, Sigma-Aldrich), mouse monoclonal anti-β-tubulin (1∶2000; IgG_1_, clone D66, Sigma-Aldrich), rabbit polyclonal anti-pericentrin (1∶1000, Abcam), mouse monoclonal anti-γ-tubulin (1∶1000; Sigma-Aldrich), rabbit polyclonal anti-γ-tubulin (1∶1000; Sigma-Aldrich), mouse monoclonal anti-tau-1 (1∶1000; IgG_2a_, clone PC1C6, Chemicon International, Temecula, CA, USA), mouse monoclonal anti-MAP2 (1∶1000; IgG_1_, clone HM-2, Sigma-Aldrich), rabbit polyclonal anti-PLCγ1 (Cell Signaling Technology, Danvers, MA, USA), and mouse monoclonal anti-ankyrin G (1∶1000; IgG_1_, Life Technologies Invitrogen, Grand Island, NY, USA). The following Alexa Fluor secondary antibodies were obtained from Life Technologies Molecular Probes: goat anti-mouse IgG 488, goat anti-mouse IgG 546, goat anti-mouse IgG 633, goat anti-rabbit IgG 488, goat anti-rabbit IgG 546, goat anti-rabbit IgG 633, donkey anti-goat IgG 546, 647, and 488, donkey anti-rabbit IgG 488, donkey anti-mouse IgG 488 and 565, goat anti-mouse IgG_1_ 488, and goat anti-mouse IgG_2a_ 488 and 546. DAPI (1∶1000) was from Life Technologies Molecular Probes.

### Cell and neuronal culture and transfections

HeLa cells (ATCC CCL-2) and hTERT-RPE1 cells (ATCC) were maintained in Dulbecco's modified Eagle's medium (DMEM) containing 10% fetal bovine serum. All constructs were transfected using GenJet Plus (SignaGen Laboratories, Rockville, MD, USA) 48 hours after transfection according to the manufacturer's instructions. For immunoblotting studies, cell lysates were prepared on ice with 2% Triton X-100/protease inhibitor cocktail [28]. Proteins were quantified using the BCA assay, with BSA as the standard (Thermo Scientific, Waltham, MA, USA). For immunofluorescence experiments, HeLa cells were grown on flamed coverslips and fixed with cold methanol for 4 min at −20°C. Cells were then washed in PBS for 10 min and blocked in 10% goat serum/PBS/0.2% Triton X-100 for one hour. Cells on coverslips were then incubated with primary antibodies for 2 hours at room temperature and then the appropriate secondary antibodies for another 2 hours. To visualize nuclei, cells were stained using DAPI. Cells were mounted with Gel/Mount (BioMeda, Foster City, CA, USA). For quantitation of centrosomal proteins, the fluorescence intensity of the region of interest was measured. At least 100 cells on each of 3 coverslips were evaluated per experimental condition. Image processing and measurements were carried out using NIH ImageJ software.

Primary cultures of rat cerebral cortical neurons were prepared from E18 embryos obtained from timed pregnant Sprague-Dawley rats (Taconic Biosciences, Hudson, NY, USA) narcotized with CO_2_ (in cylinders) then decapitated using a guillotine. All animal studies were approved by the National Institute of Neurological Disorders and Stroke/National Institute on Deafness and Other Communication Disorders Animal Care and Use Committee (Protocol 1151–12). Neurons were transfected with spastin and NA14 constructs using the Amaxa Rat Neuron Nucleofector Kit, program 0–03, according to the manufacturer's protocol (Lonza Group, Basel, Switzerland). Neurons were then plated at a density of ∼2.6×10^4^/cm^2^ on cover slips and maintained as described previously [30]. At 3 days *in vitro* (DIV) and 6DIV, neurons were fixed with methanol or paraformaldehyde, immunostained with primary and Alexa Fluor secondary antibodies, mounted, and imaged using a Zeiss LSM710 laser-scanning confocal microscope. Primary antibodies against the following proteins were used: spastin, NA14, pericentrin, γ-tubulin, MAP2, tau-1, and β-tubulin.

### Stable cell lines

Stable cell lines were prepared as previously described [31]. Briefly, HEK293T cells (ATCC) were co-transfected with pCMV-dR8.2 dvpr, pCMV-VSV-G (Addgene) and the respective pCDH-CMV-MCS-EF1-Puro-based constructs to generate lentivirus for NA14 expressing-cell lines. For shRNA-expressing cell lines, pLKO.1 Puro-based constructs were used instead.

### Immunoprecipitation

HeLa cells co-transfected with untagged NA14 and Myc-spastin, or else transfected with untagged NA14 alone or Myc-spastin alone, were washed twice with PBS, harvested in 0.5% Triton X-100/PBS, and clarified by centrifugation at 130,000×*g* for 30 min. Extracts (100 µg of protein) were incubated for 1–2 hours at 4°C with 5 µg of rabbit polyclonal anti-SSNA1 antibodies or 5 µg of mouse monoclonal anti-c-Myc antibodies. Protein A/G PLUS-agarose beads (Santa Cruz Biotechnology) were added for incubation overnight at 4°C. Beads were subsequently washed three times with 0.5% Triton X-100/PBS. Bound proteins were resolved by SDS-PAGE and immunoblotted with mouse monoclonal anti-c-Myc antibodies, rabbit polyclonal anti-SSNA1 antibodies, mouse polyclonal anti-SSNA1 antibodies, and goat anti-Myc antibodies.

### Statistics

Statistical analyses were performed using Student's *t* test, assuming unequal variance. *P*<0.05 was considered significant.

## Results

### NA14 localizes to the centrosome

**Figure 1:**
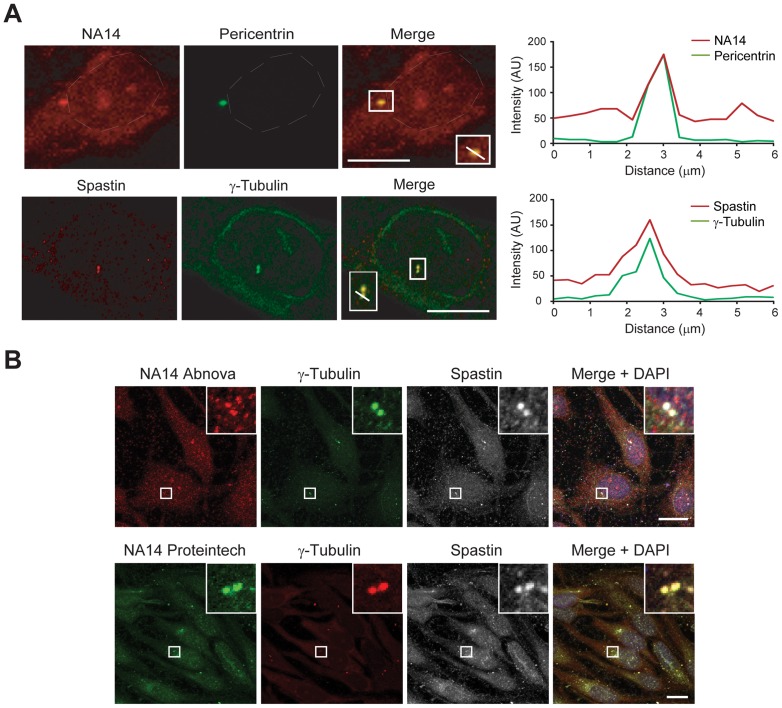
Endogenous NA14 and spastin proteins localize to the centrosome. (A) Endogenous NA14 and spastin (red) co-localize at the centrosome in HeLa cells, which were co-stained for the centrosomal markers pericentrin or γ-tubulin (green). Merged images are at the right, and boxed areas are enlarged in the insets. Images were acquired using confocal immunofluorescence microscopy, and relative fluorescence intensities for the indicated linear regions in the merged images, measured using Zeiss LSM710 software, are graphed. Note the high degree of line-scan overlap (right). AU, arbitrary units. (B) HeLa cells co-immunostained for NA14 (two different antibodies), spastin and γ-tubulin are shown. Merged images with DAPI nuclear staining are at the right. Boxed areas are enlarged in the upper right-hand corner insets. Scale bar: 10 µm.

It has been previously shown that spastin and NA14 interact with one another [14]. We compared their cellular distributions in HeLa cells using specific markers of the centrosome (pericentrin and γ-tubulin), with visualization using confocal immunofluorescence microscopy. NA14 and spastin both localize to the centrosome in HeLa and RPE1 cells (Fig. 1A and Fig. S1). To confirm their co-localization, we co-stained NA14 (using two different commercial antibodies) and spastin in HeLa cells along with γ-tubulin (Fig. 1B).

### Spastin M87 localizes to the centrosome and NA14 selectively to the centrioles

**Figure 2:**
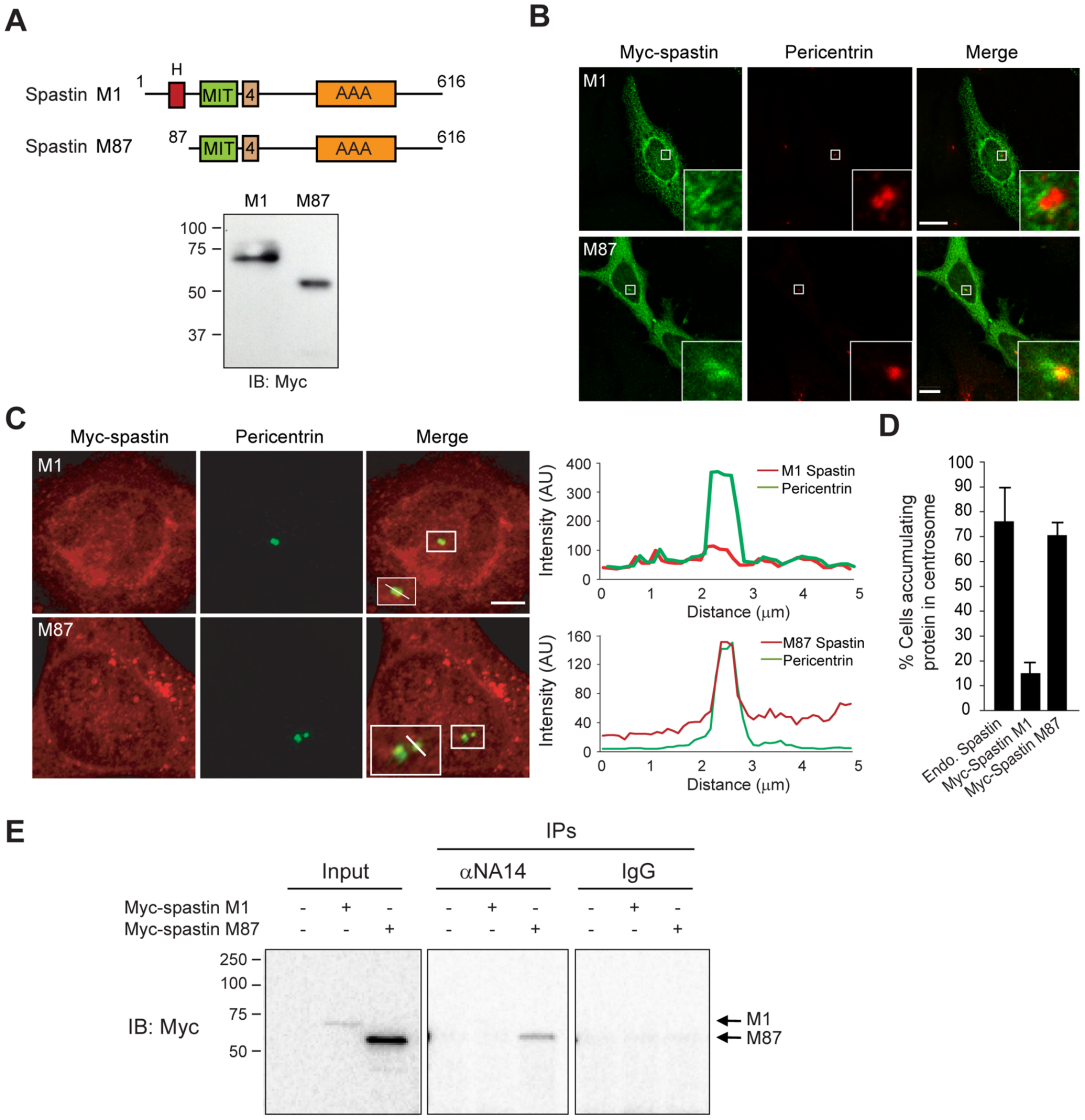
NA14 localizes to the centrosome and interacts with the M87 isoform of spastin. (A) Top, Schematic diagrams showing the domain organizations of spastin isoforms generated through use of 2 different translation start codons (exon 4 splice cassettes are also indicated). Bottom, HeLa cells were transfected with Myc-tagged spastin M1 or spastin M87 and immunoblotted (IB) with Myc-tag antibody. Migrations of molecular weight standards (in kDa) are indicated at the left. (B) Myc-spastin isoforms M1 and M87 (green) were co-stained with pericentrin antibodies (red) and visualized by confocal microscopy. Scale bars: 10 µm. (C) Myc-spastin isoforms M1 and M87 (red) were co-stained with pericentrin antibodies (green) and visualized by confocal microscopy. Relative fluorescence intensities for the indicated linear regions in merged images (boxed areas enlarged in insets at the bottom left) were measured using Zeiss LSM710 software and graphed (right). Scale bar: 10 µm. (D) Relative numbers of HeLa cells accumulating the indicated endogenous or recombinant spastin proteins in the centrosome were quantified (means ±SEM; *n* = 3, with 100 cells per trial). (E) HeLa cells were transfected with Myc–spastin M1 or M87 as indicated, immunoprecipitated (IP) with NA14 antibodies or non-immune IgG, and immunoblotted (IB) with Myc-epitope antibodies. The input represents 5% of the starting material. Migrations of molecular weight standards (in kDa) are indicated at the left, and the spastin isoform to the right.

The interaction between NA14 and spastin was originally identified by yeast two-hybrid screening, and a fragment of NA14 (residues 33–119) lacking the N-terminal coiled-coil domain implicated in interactions with microtubules is sufficient to bind spastin [14]. We used immunofluorescence and co-immunoprecipitation studies to identify the isoform of spastin interacting with NA14 (Fig. 2). In immunofluorescence localization experiments, endogenous spastin and recombinant Myc-spastin M87 accumulated at the centrosome, while Myc-spastin M1 did not when expressed at similar levels (Fig. 2A–D). At these levels of expression, there was no significant disruption of the microtubule network (Fig. S2A). Consistent with these immunolocalization data, upon immunoprecipitation of Myc-tagged spastin M1- or M87-transfected HeLa cells, we observed that spastin M87 co-precipitated with endogenous NA14, but spastin M1 did not (Fig. 2E). One caveat is that expression levels for Myc-spastin M1 were lower than those of Myc-spastin M87.

**Figure 3:**
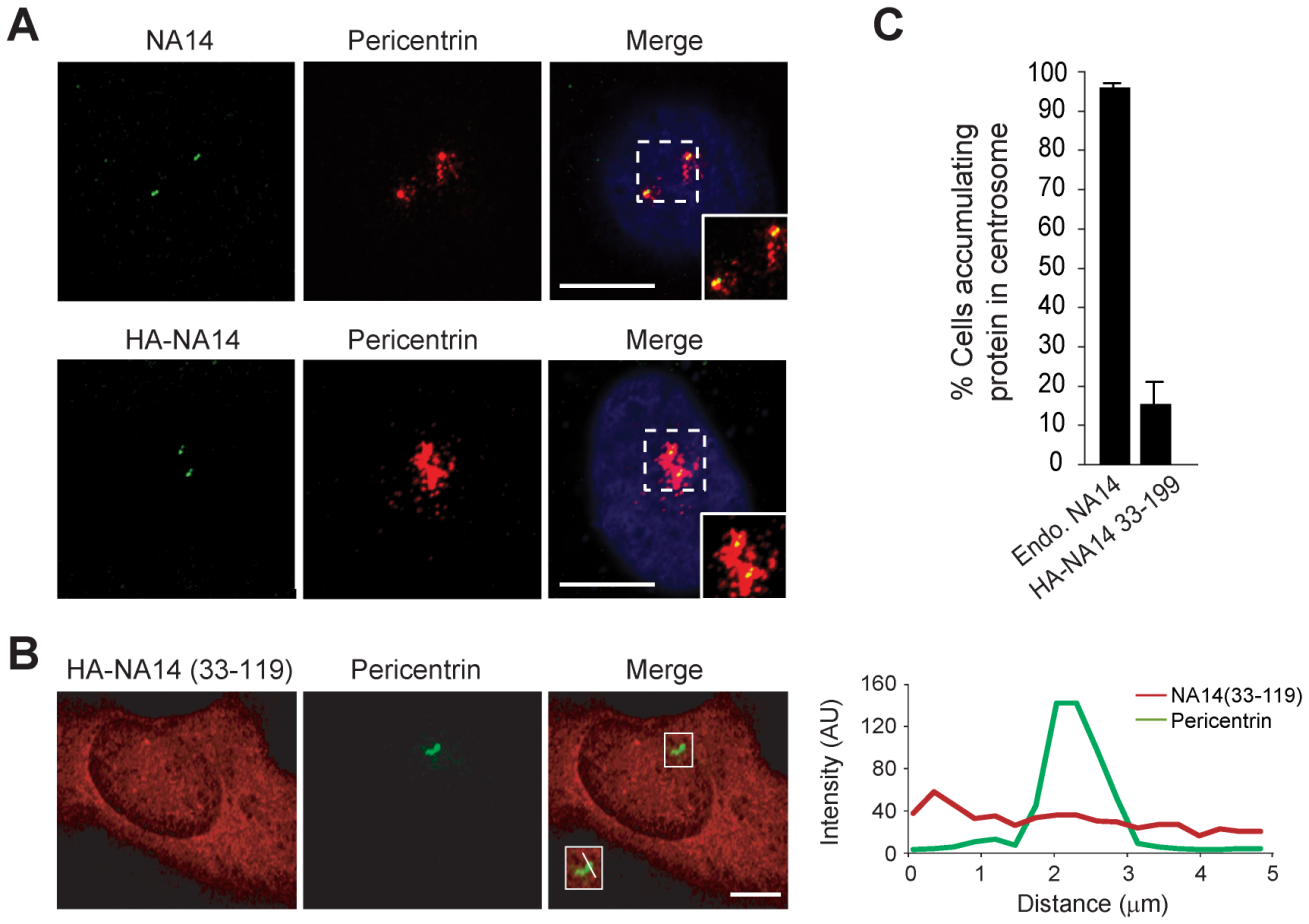
Wild-type NA14 but not NA14 (33–119) localizes to the centrosome. (A) Endogenous NA14 and HA-NA14 from stable cell lines (green) were co-stained with pericentrin antibodies (red) and visualized using confocal microscopy. Hatched boxes are enlarged in the lower right-hand corner insets. (B) HA-NA14 (33–119) stably expressed in HEK293T cells (red) was co-stained with pericentrin antibodies (green). Relative fluorescence intensities for the indicated linear regions in merged images (boxed area enlarged in lower left-hand corner inset) were measured using Zeiss LSM710 software and graphed. AU, arbitrary units. Scale bars: 10 µm. (C) Relative numbers of HeLa cells accumulating the indicated endogenous or recombinant NA14 proteins in the centrosome were quantified (means ±SEM; *n* = 3, with 100 cells per trial).

The centrosome is composed of a pair of centrioles and the pericentriolar material. Endogenous and wild-type recombinant NA14 localized to the centrosome, in particular at the centrioles (Fig. 3A). However, the NA14 (33–119) fragment did not accumulate at the centrosome (Fig. 3B and C). These data suggest that spastin M87 and NA14 interact in the centrosome and that the first 32 amino acid residues of NA14 are crucial for the localization of NA14 to centrioles.

### Both NA14 and spastin are involved in cytokinesis

**Figure 4:**
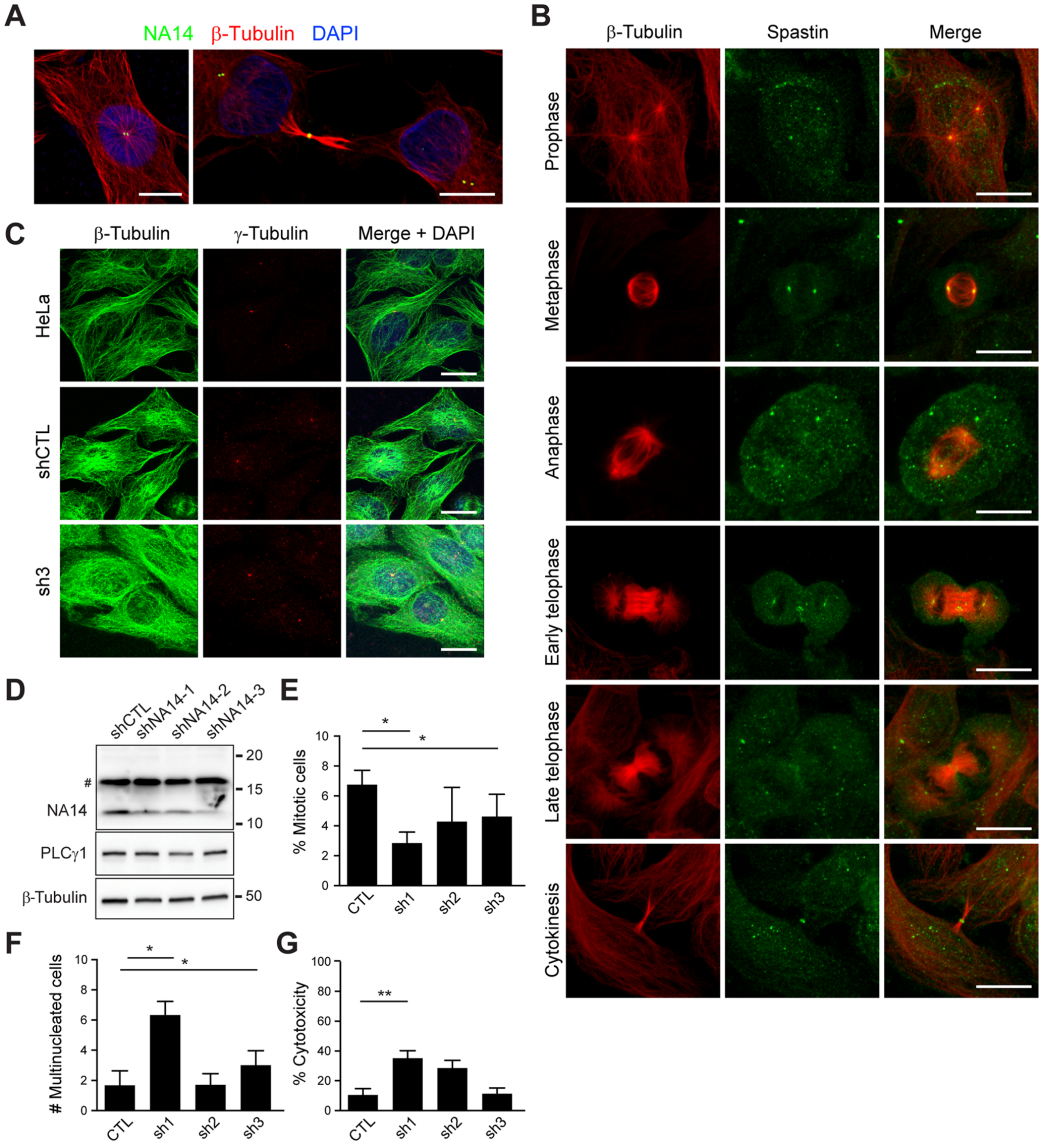
NA14 and spastin localize to the centrosome and midbody during cytokinesis. (A) Merged images of endogenous NA14 (green) accumulated at the centrosome during interphase and at the midbodies during late cytokinesis, along with β-tubulin (red). (B) Endogenous spastin (green) localizes to the centrosome and the midbodies, as shown by co-staining for β-tubulin (red). Merged images are at the right. (C) HeLa cell lines stably expressing control shRNA (shCTL) or shRNAs against NA14 (sh3 shown) were immunostained for endogenous β-tubulin (green) and γ-tubulin (red), with merged images at the right. Bar: 10 µm. (D) Cell extracts from cell lines stably expressing the indicated shRNAs were immunoblotted for NA14. PLCγ1 (149 kDa) and β-tubulin levels were monitored to control for protein loading. # denotes a cross-reacting band. (E and F) Mitotic and multinucleated cells were quantified in control and NA14 shRNA stable cell lines (means ±SEM; *n* = 3, with 100 cells per experiment). Nuclei were identified by co-staining with DAPI. (G) Quantification of cell death in control and NA14 shRNA stable cells lines by measuring lactate dehydrogenase release from cells (means ±SEM; *n* = 3, with 100 cells per experiment). *p<0.05; **p<0.01.

To assess the functional importance for the interaction of NA14 and spastin in cells, we established stable cell lines expressing HA-tagged NA14 or else shRNAs against NA14. We then examined the distributions of NA14 and spastin in HeLa cells during different stages of the cell cycle. HA-NA14 localized to centrioles and also to the midbody (Fig. 4A), a transient structure generated between the two daughter cells in the final stages of cell division. A midbody contains bundles of microtubules and various proteins, including ESCRT components and centrosomal proteins such as CEP55. Endogenous spastin is also found within the centrosome during the cell cycle, with this distribution particularly evident from prophase through anaphase (Fig. 4B). Consistent with previous reports [12], [14], [29], [32], spastin similarly localized to midbodies during the abscission phase late in cytokinesis (Fig. 4B).

Given the physical interaction of NA14 and spastin as well as their similar distributions at the centrosome and midbodies during the cell cycle, we examined the role of NA14 in cell division. We generated three different cell lines stably expressing shRNAs to deplete NA14 and compared them to control cell lines. While the level of expression of NA14 was significantly reduced in the specific shRNA cell lines, the overall appearance of the cells and the centrosomes appeared unchanged (Fig. 4C and D). However, in cells depleted of NA14, in particular the sh1 line, there was a statistically significant reduction in the percentage of mitotic cells as compared to control cells (Fig. 4E). Consistent with this impairment in cytokinesis, NA14 depletion caused significant increases in the number of multinucleated cells in two of the three lines, with cells accumulating an average of ∼6 nuclei (Fig. 4F). This was most prominent in the sh1 line, and in fact levels of cell death were also elevated in this line, suggesting that there may be cytotoxic effects of NA14 depletion (Fig. 4G).

The importance of microtubules in the accumulation of centrosomal proteins was examined using a microtubule regrowth assay (Fig. S2B). In this assay, the microtubule network is first disrupted by cold treatment, and then allowed to re-form after incubation in warm media (Fig. S2B). Although levels of NA14 and spastin expression were modified by overexpression and/or depletion, the recovering microtubules grew in the warm medium in a manner similar to that in control cells (Figs. S2B and S3).

### NA14 localizes to the centrosome of neuronal cells and enhances axon development

**Figure 5:**
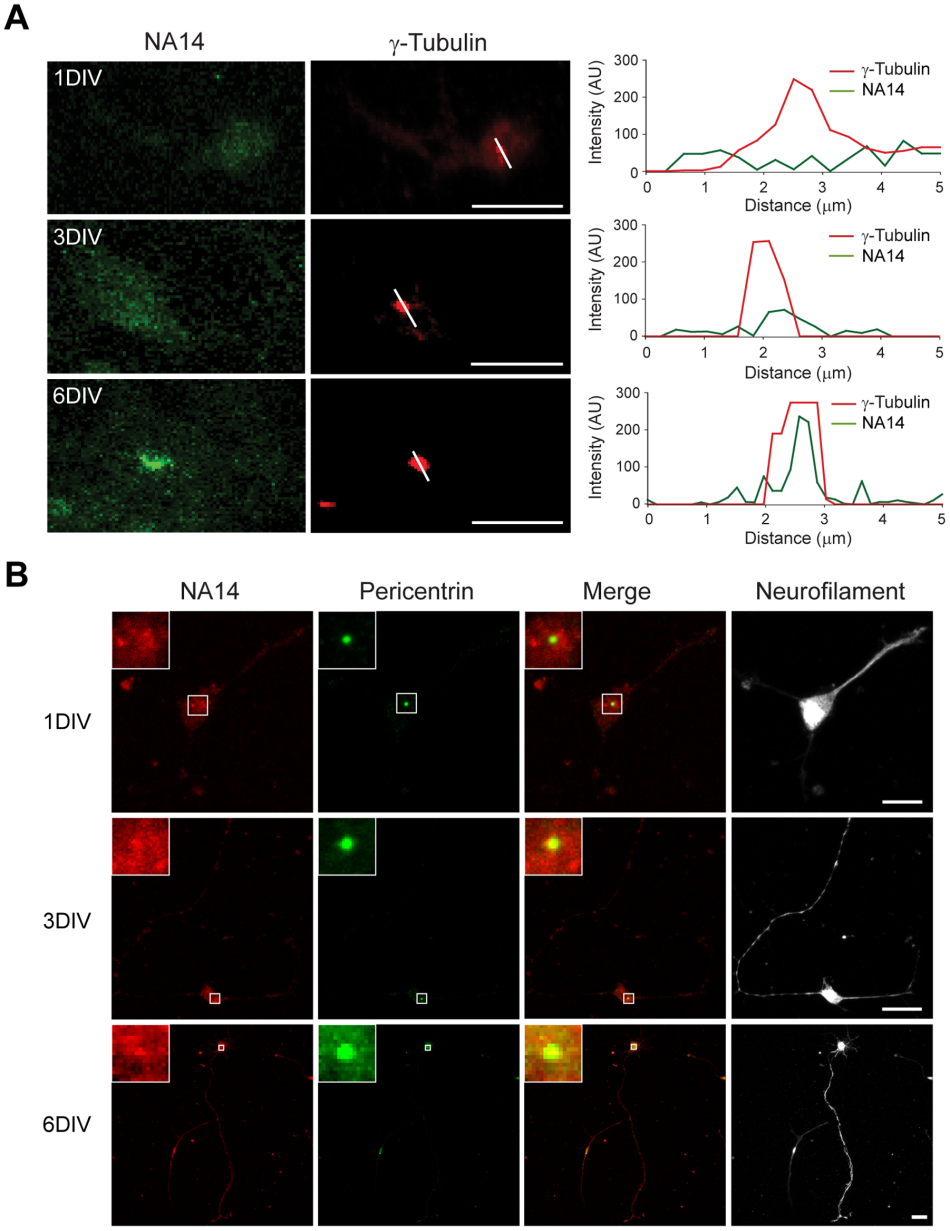
NA14 accumulates at the base of axons in cultured cortical neurons at 6DIV. (A) Mixed cortical neurons at 1, 3 and 6DIV were immunostained for endogenous NA14 (green) and γ-tubulin (red); NA14 localizes to the centrosome at 6DIV. Scale bars: 40 µm. Relative fluorescence intensities for the indicated linear regions in merged images were measured using Zeiss LSM710 software and graphed (at the right). AU, arbitrary units. (B) Neurons at 1, 3 and 6DIV were immunostained for endogenous NA14 (red), neurofilaments (gray) and pericentrin (green). The merged images are indicated. Scale bars: 40 µm.

Spastin is mutated in patients with SPG4, the most common form of HSP. Since a number of mammalian cell culture models for HSPs have exhibited alterations in neuronal morphology, particularly axon elongation and branching [1], [30], [33]–[38], we assessed the role of NA14 in neuronal development. We immunostained rat cerebral cortical neurons in primary culture that had been transfected with NA14 constructs for tau-1 (axonal marker), MAP2 (dendritic marker), and γ-tubulin (centrosome marker) to facilitate assessment (Fig. S4). NA14 accumulated in the centrosome at 6DIV (Fig. 5A), when the centrosome is positioned early at the base of the future axon to initiate its outgrowth (Fig. 5B).

**Figure 6:**
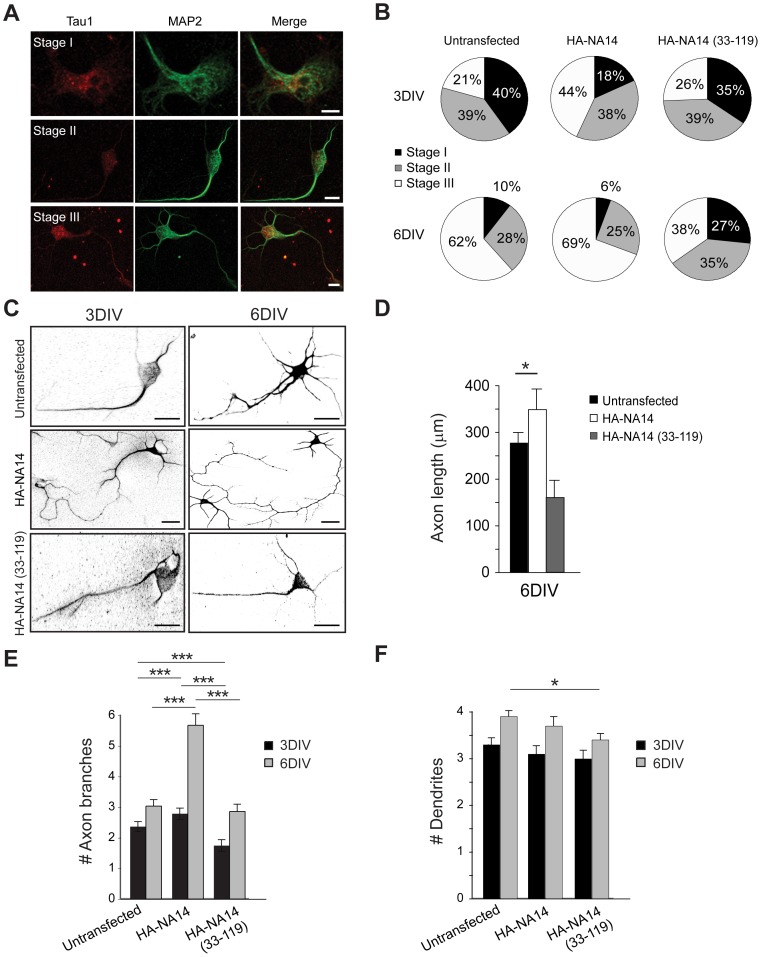
Morphological analysis of control and NA14-overexpressing cortical neurons. (A) Representative neurons at developmental stages I, II, and III were co-stained for tau-1 (red) and MAP-2 (green). Merged images are at the right. Scale bar: 40 µm. (B) Pie graphs showing the percentages of cortical neurons in stages I, II, and III in each experimental group (*n*>100). More neurons remained in stages I and II in untransfected and NA14 (33–119) expressing cultures than in HA-NA14 expressing neurons. (C) β-tubulin staining (black) reveals processes of transfected and control cultured neurons at 3 and 6DIV. Scale bar: 40 µm. (D and E) Quantifications of primary axon length as well as number of primary axon branches in cortical neurons in primary culture (means ±SEM; *n* = 3, with 30–60 neurons per trial). (F) Numbers of dendrites per cell are shown graphically (means ±SD; *n* = 3, with 30–60 neurons per trial). *p<0.05, ***p<0.001.

Since spastin plays a crucial role in axon development, we assessed the potential role of the interaction of spastin with NA14, expressing full-length and deletion mutants of NA14 and counting the numbers of cells in each stage of development. We considered three stages of neuronal development. Shortly after they adhere to the substrate, neurons extend a broad lamellipodium (stage I) that subsequently coalesces into multiple immature “minor” processes (stage II). One of these minor processes then becomes the axon, usually by the second day after plating, and the others develop into dendrites (stage III; Fig. 6A). Visual observations of cell form revealed close similarities among transfected and control neurons. However, axon length and primary branching were significantly greater in neurons expressing NA14 as compared to controls and neurons expressing the NA14 (33–119) mutant (Fig. 6B). NA14 seemed to accelerate the development of the cultured neurons, while expression of the NA14 (33–119) mutant was inhibitory. Furthermore, cells expressing wild-type NA14 showed a significant increase in axon length and branching at 6DIV as compared to control and NA14 (33–119)-expressing cells (Fig. 6C–E). Conversely, overexpression of the NA14 (33–119) mutant inhibited axon outgrowth (Fig. 6D). Finally, we analyzed the number of dendrites using MAP2 immunostaining. We observed that dendrite number was not altered by wild-type NA14 overexpression, but it was slightly decreased at 6DIV in cells overexpressing NA14 (33–119) (Fig. 6F).

## Discussion

In this study, we have demonstrated that the spastin M87 isoform co-localizes with its interacting partner NA14 at the centrosome, a non-membranous organelle composed of centrioles and pericentriolar material [21], [22]. An association between NA14 and the spastin M1 isoform cannot be formally ruled out, however, as the lower expression of Myc-tagged spastin M1 could have affected the ability of our immunoprecipitation experiments to detect an interaction. Evaluation of HeLa cell lines stably expressing epitope-tagged NA14 showed that NA14 is enriched at centrioles. Over the years, several groups have reported the localization of NA14 and its orthologs to the centrosome, first in the biflagellated unicellular organism *C. reinhardtii* [18]. The *C. reinhardtii* NA14 ortholog DIP13 localizes to cytoplasmic microtubules and basal bodies, which are structurally and functionally very similar to mammalian centrioles. Several other studies using immunostaining and mass spectrometry-based proteomic analyses have supported the localization of NA14 to the centrosome [17], [38]. Even so, the function of NA14 at the centrosome remains unknown.

A number of studies have suggested that DIP13/NA14 is involved in cell division by stabilizing microtubules or linking microtubule structures to the division machinery [18], [39]. Indeed, knockdown of *C. reinhardtii* DIP13 results in multinucleated and multiflagellate cells. Our data suggest that the depletion of NA14 does not affect the structure of the centrosome *per se* but might stabilize microtubules, in particular during axon development. Also, our data indicate that NA14 is directly involved in cell division and could be important for abscission, the final phase of cytokinesis. In concert with previous studies, these observations suggest that NA14 is a molecular adaptor involved in targeting proteins to the centrosome and midbodies.

In agreement with previous observations [18], [39], we found that NA14 localizes to midbodies during cytokinesis. This highly-regulated process between two daughter cells requires a large variety of molecules, including the NA14-interacting protein spastin that is also highly enriched at midbodies [29], [40]. Thus, NA14 could conceivably play a role in cytokinesis by regulating the localization and the microtubule-severing activity of spastin at the midbodies. Of possible relevance, NA14 expression is up-regulated in T-cell acute lymphoblastic leukemia characterized by an amplification of 9q34 [41] and is differentially expressed in pancreatic cancer [42].

Several groups have shown that spastin influences microtubule dynamics in growth cones, regulating the stability of axons and axonal transport [30], [33]–[38], [43]–[45]. For example, Yu *et al.* [44] showed that expression of spastin regulates axon length and number of branches. They further observed that the expression of spastin positively correlates with the formation of branches and the axon size [44]. Based on our studies in neurons, it seems reasonable to postulate that NA14 might regulate spastin. Notably, NA14 is accumulated at the centrosome during the initiation of axon formation. Spastin and related proteins such as katanin have been implicated in releasing microtubules from the centrosome during mitosis, as well as in mechanisms that regulate microtubule length in axons of postmitotic neurons [46], [47]. Thus, we can imagine that NA14 might trigger spastin-dependent microtubule-severing in a specific cellular location (i.e., at the centrosome of neurons) at a specific time (i.e., 6DIV). Further studies of the NA14-spastin interaction *in vitro* and in cells will be necessary to test this hypothesis.

Long axons of neurons within the corticospinal tract are highly dependent on spastin function. NA14 seems to play a role in neuronal development, in particular in axon outgrowth. The accumulation of NA14 could thus regulate the recruitment and/or regulation of spastin during the development of axons. In fact, previous studies have shown that spastin acts as a microtubule-severing protein in mammalian cells, and expression of spastin mutants unable to hydrolyze ATP result in the increased formation of stable bundles of microtubules [9]. Moreover, Rodríguez-Rodríguez *et al.* [48] have shown that NA14 can create a dynamic matrix between microtubules and spastin, providing a scaffold for anchoring proteins that are involved in microtubule nucleation and axonal development. A transport role for NA14 also has precedent, since NA14 has previously been implicated in transport of the orphan receptor TPRA40/GPR175, and the interaction with NA14 is required for the effects of TPRA40/GPR175 on cell division in mouse embryos [49]. Lastly, NA14 was identified in a high-content screen for cilia genes as a protein involved in transport/trafficking of ciliary proteins [50].

In conclusion, our findings suggest that NA14 is involved in cytokinesis and neuronal development. NA14 could regulate the localization and activity of the microtubule-severing AAA protein spastin in the midbody as well as during axon outgrowth. The involvement of NA14 in dynamic remodeling of the microtubule cytoskeleton, in developing and adult axons, and in the regulation of spastin serves a springboard to understanding the functional roles of their interaction, particularly in the pathogenesis of HSP.

## Supporting Information

Figure S1 NA14 and spastin localize to the centrosome in RPE1 cells. Top panels, NA14 (red) was visualized along with β-tubulin (green). Lower panels, Spastin (green) and pericentrin (red) were co-immunostained. DAPI (blue) stains the nuclei. Merged images are at the right. Scale bars: 10 µm.
(TIF)

Figure S2 Effects of spastin depletion or overexpression on the microtubule network. (A) HeLa cells were transfected with Myc-Spastin M1 or M87 as indicated, and then immunostained for β-tubulin (red) or Myc-epitope (green). (B) To assess effects on microtubule nucleation, HeLa cells were transfected with Myc-spastin M87 or siRNA against spastin, with control cells left untransfected. After 2 days, cells were incubated on ice for 60 min to disassemble the microtubule network, and then incubated in warm media for the indicated time periods (Recovery) before fixation. Then, cells were co-immunostained for β-tubulin (red, middle; green, top and bottom) and pericentrin (red, top and bottom; green, middle). DAPI nuclear staining is blue. Scale bars: 10 µm.
(TIF)

Figure S3 Effects of NA14 depletion on microtubule nucleation. HeLa cell lines stably-expressing the indicated NA14-specific (sh1-3) or control (shCTL) shRNAs were incubated on ice for 60 min to disassemble the microtubule network, then incubated in warm media for the indicated time periods (Recovery) before fixation. Next, cells were co-immunostained for β-tubulin (green) and pericentrin (red). DAPI nuclear staining is blue. Scale bars: 10 µm.
(TIF)

Figure S4 Expression of recombinant NA14 in cultured cortical neurons. Mixed cortical neurons were transfected with HA-NA14 or HA-NA14 (33-119). Representative neurons at 3DIV were co-stained for tau-1 (green), MAP-2 (red) and HA-epitope (blue). Scale bars: 40 µm.
(TIF)
