# Supplementary figures and images for: Spastin-Interacting Protein NA14/SSNA1 Functions in Cytokinesis and Axon Development
Source: PLoS One. 2014 Nov 12;9(11):e112428. doi: 10.1371/journal.pone.0112428 (PMC4229207; doi:10.1371/journal.pone.0112428)

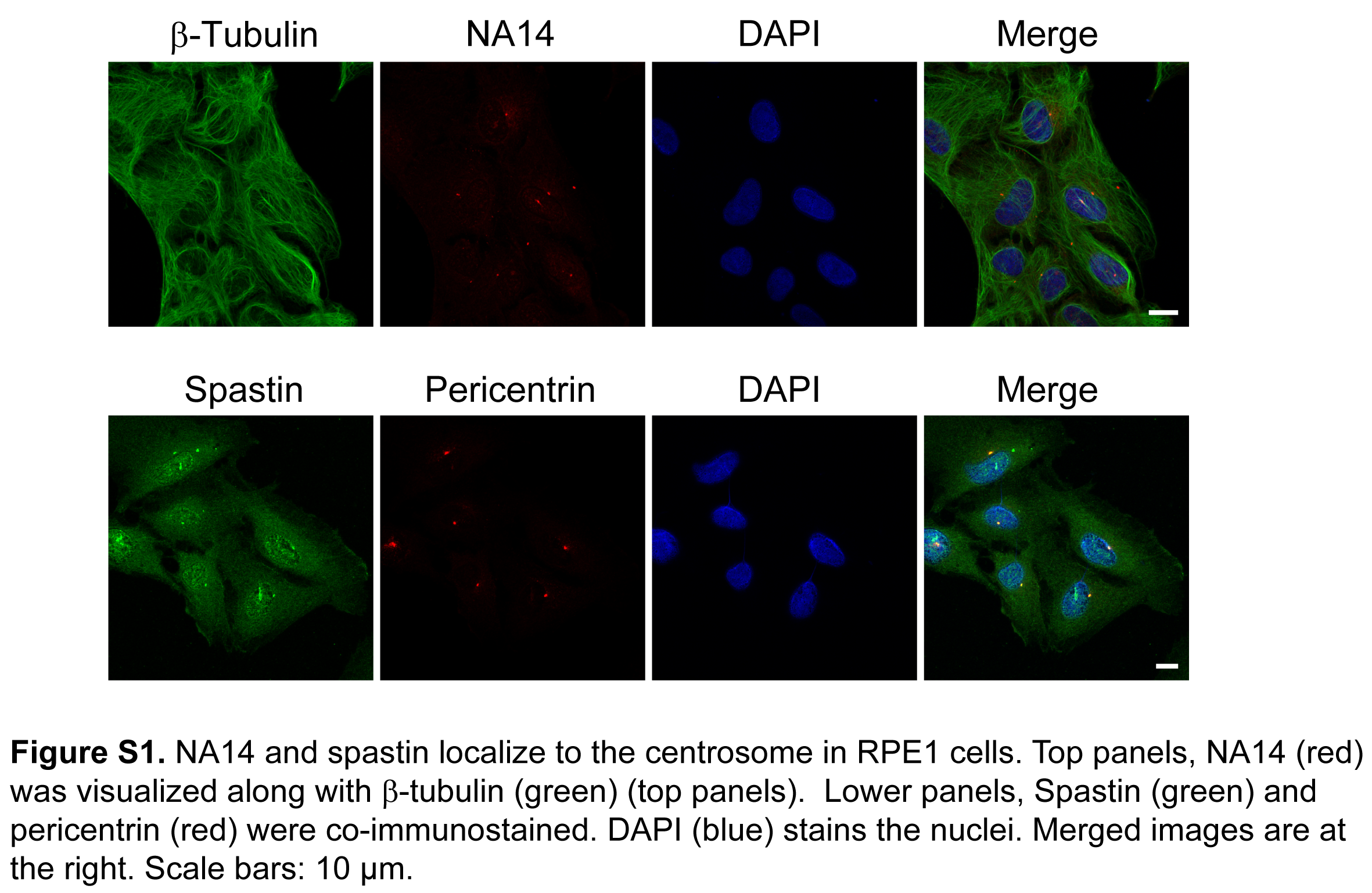

Supplement: Figure S1 — NA14 and spastin localize to the centrosome in RPE1 cells. Top panels, NA14 (red) was visualized along with β-tubulin (green). Lower panels, Spastin (green) and pericentrin (red) were co-immunostained. DAPI (blue) stains the nuclei. Merged images are at the right. Scale bars: 10 µm. (TIF) [file pone.0112428.s001.tif]

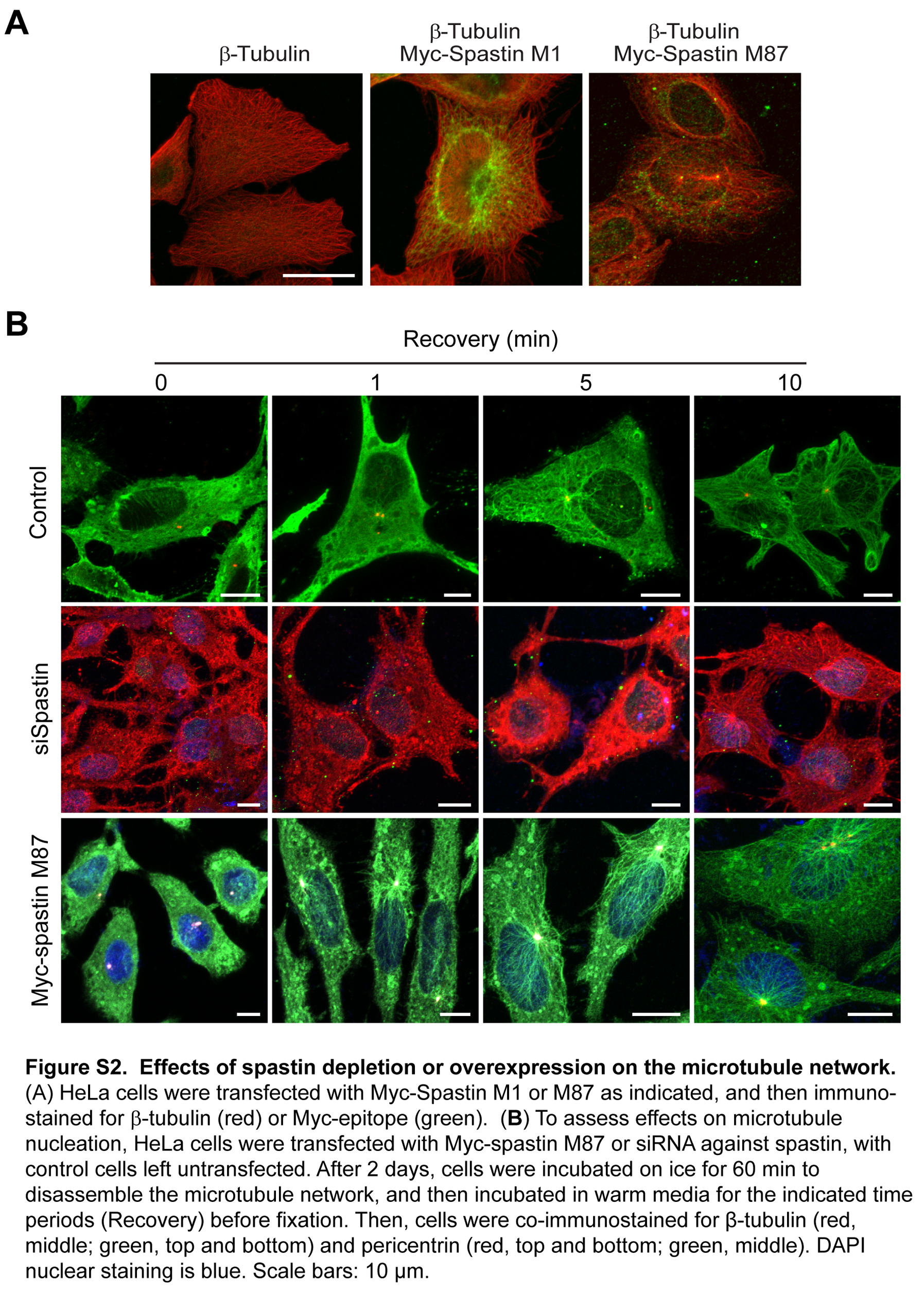

Supplement: Figure S2 — Effects of spastin depletion or overexpression on the microtubule network. (A) HeLa cells were transfected with Myc-Spastin M1 or M87 as indicated, and then immunostained for β-tubulin (red) or Myc-epitope (green). (B) To assess effects on microtubule nucleation, HeLa cells were transfected with Myc-spastin M87 or siRNA against spastin, with control cells left untransfected. After 2 days, cells were incubated on ice for 60 min to disassemble the microtubule network, and then incubated in warm media for the indicated time periods (Recovery) before fixation. Then, cells were co-immunostained for β-tubulin (red, middle; green, top and bottom) and pericentrin (red, top and bottom; green, middle). DAPI nuclear staining is blue. Scale bars: 10 µm. (TIF) [file pone.0112428.s002.tif]

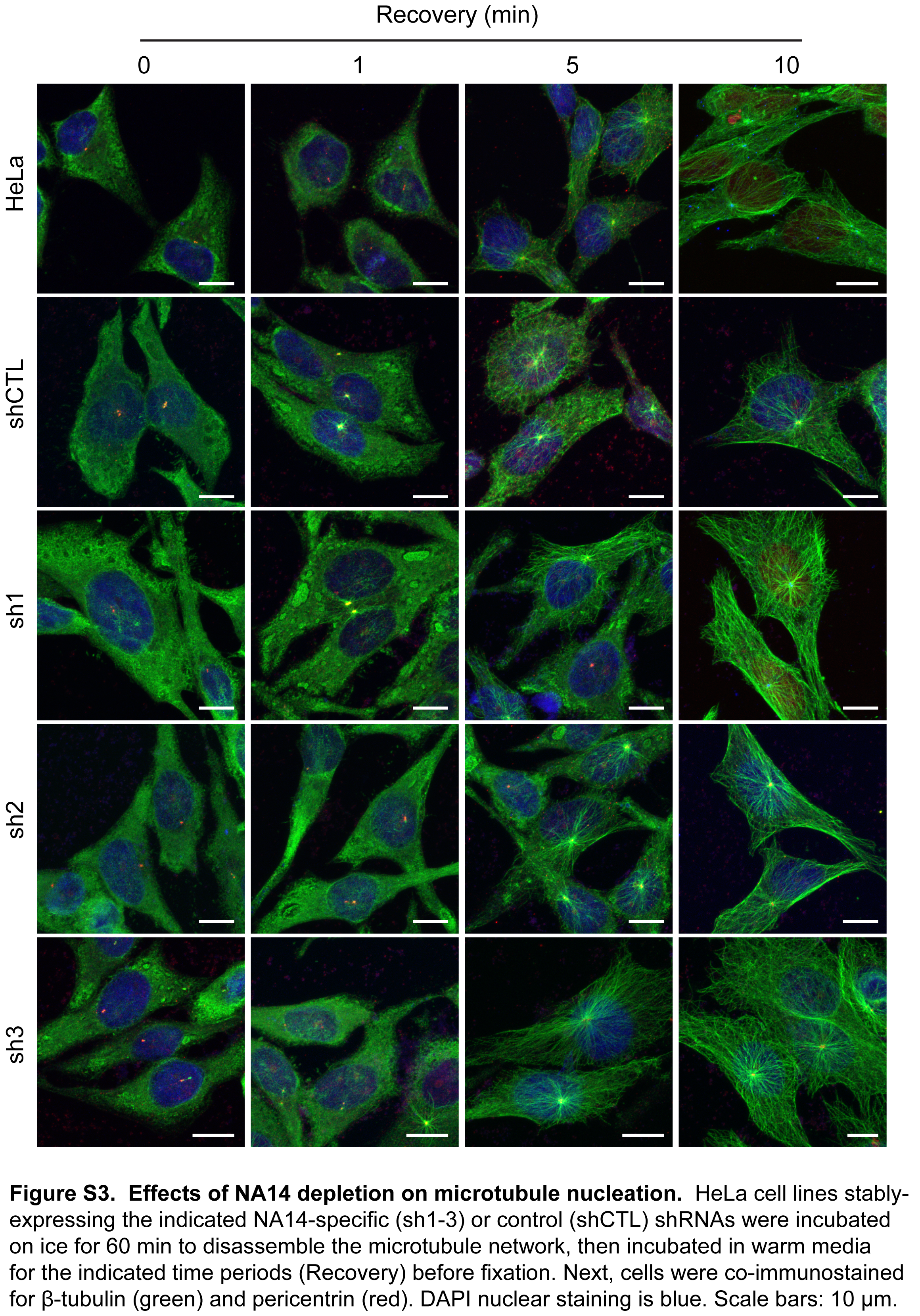

Supplement: Figure S3 — Effects of NA14 depletion on microtubule nucleation. HeLa cell lines stably-expressing the indicated NA14-specific (sh1-3) or control (shCTL) shRNAs were incubated on ice for 60 min to disassemble the microtubule network, then incubated in warm media for the indicated time periods (Recovery) before fixation. Next, cells were co-immunostained for β-tubulin (green) and pericentrin (red). DAPI nuclear staining is blue. Scale bars: 10 µm. (TIF) [file pone.0112428.s003.tif]

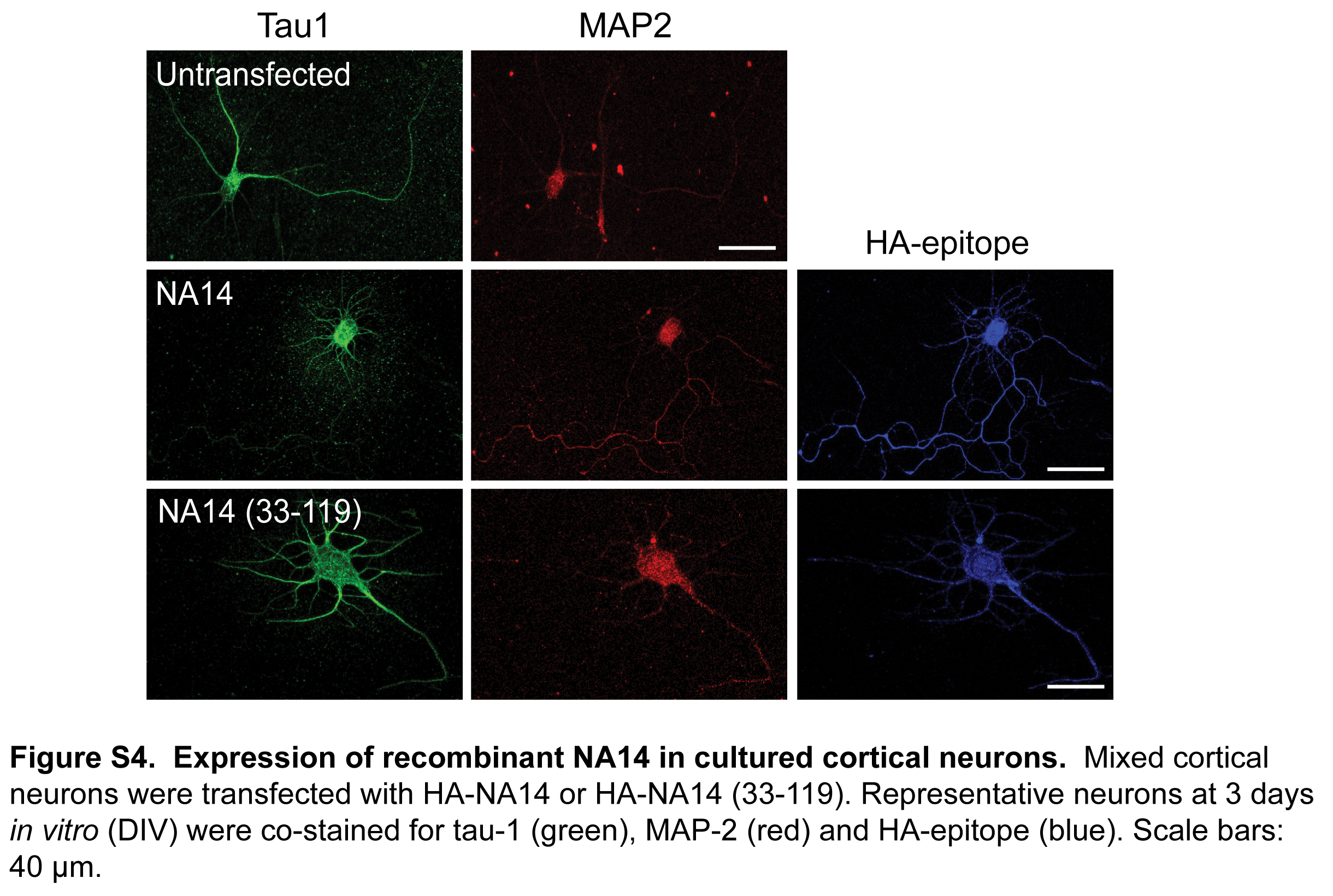

Supplement: Figure S4 — Expression of recombinant NA14 in cultured cortical neurons. Mixed cortical neurons were transfected with HA-NA14 or HA-NA14 (33-119). Representative neurons at 3DIV were co-stained for tau-1 (green), MAP-2 (red) and HA-epitope (blue). Scale bars: 40 µm. (TIF) [file pone.0112428.s004.tif]
